# Review of Research Status and Development Trends of Wireless Passive LC Resonant Sensors for Harsh Environments

**DOI:** 10.3390/s150613097

**Published:** 2015-06-04

**Authors:** Chen Li, Qiulin Tan, Pinggang Jia, Wendong Zhang, Jun Liu, Chenyang Xue, Jijun Xiong

**Affiliations:** 1 Key Laboratory of Instrumentation Science & Dynamic Measurement, Ministry of Education, North University of China, Tai Yuan 030051, China; E-Mails: flanklichen@163.com (C.L); wdzhang@nuc.edu.cn (W.Z.); liuj@nuc.edu.cn (J.L.); 2 Science and Technology on Electronic Test & Measurement Laboratory, North University of China, Tai Yuan 030051, China; E-Mails: pgjia@nuc.edu.cn (P.J.); xuechenyang@nuc.edu.cn (C.X)

**Keywords:** passive LC sensor, mutual inductance coupling, noncontact wireless measurement, harsh environments

## Abstract

Measurement technology for various key parameters in harsh environments (e.g., high-temperature and biomedical applications) continues to be limited. Wireless passive LC resonant sensors offer long service life and can be suitable for harsh environments because they can transmit signals without battery power or wired connections. Consequently, these devices have become the focus of many current research studies. This paper addresses recent research, key technologies, and practical applications relative to passive LC sensors used to monitor temperature, pressure, humidity, and harmful gases in harsh environments. The advantages and disadvantages of various sensor types are discussed, and prospects and challenges for future development of these sensors are presented.

## 1. Introduction

**Figure 1:**
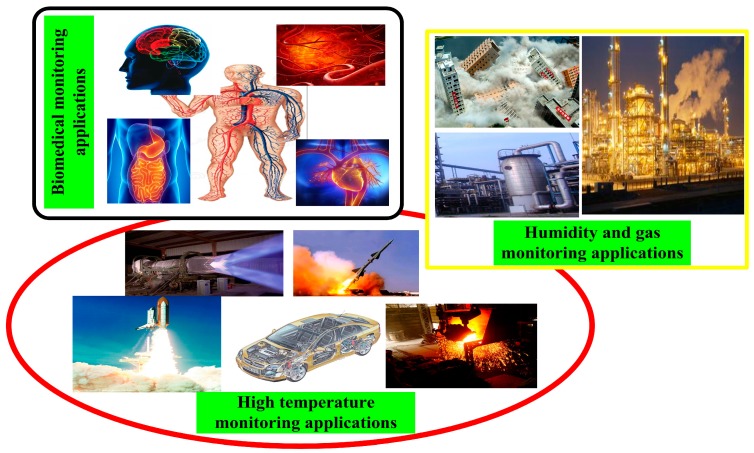
Examples of high-temperature, biomedical, and humidity and gas monitoring applications in harsh environments.

Harsh environments can typically be subdivided into high-temperature, biocompatible, corrosive and chemical, *etc.* In addition, wireless passive sensors used in these harsh-environment applications can be categorized as high-temperature, biocompatible, and humidity and gas sensors (Table 1). Measurements of certain key parameters (e.g., pressure, temperature, and humidity) in harsh environments have become increasingly important, especially pressure measurements in high-temperature environments. Figure 1 shows various harsh-environment sensing applications. Temperature and pressure measurements are critical for system control in high-temperature (such as in aerospace, automotive, aeronautics, and industrial) applications [1,2,3,4]. For biomedical applications, real-time pressure monitoring, e.g., in eyes, blood vessels, abdominal aortic aneurysm, or brain, is crucial for diagnosis, disease prevention, and treatment. Further, in some special industrial applications, wireless sensors are required to measure humidity or detect harmful gases. Many sensors have been extensively researched to monitor these key parameters in harsh environments, but their practical use has been limited due to such disadvantages as complex design, or dependence on battery power. Wireless sensing technology has a large potential for measurement in these harsh environments, and the development of wireless measurement technology has attracted significant interests. Typical commercial wireless passive sensors can be divided into surface acoustic wave (SAW), RF identification (RFID), intermodulation sensor, and RF-powered LC sensor. SAW sensors can be used for wireless monitoring in harsh environments. However, the piezoelectric material may limit their applications, and the highest operating frequency is limited to several gigahertz. RFID is mostly used for identification and can be used for telemetry, but the highest operating frequency is limited by the rectified power of the integrated circuit. The intermodulation read-out principle of a passive wireless sensor enables very small implantable health and fitness sensors, which can potentially be used for wireless monitoring (such as strain in bridges and building structure moisture, *etc.*). However, sensors based on this concept for harsh environments (such as high-temperature, biomedical, and other applications) have not been reported yet [5,6,7]. Wireless passive LC sensors are composed of inductance and capacitance and do not require wired connections to transmit signals. Thus, they are potential for complex applications in harsh environments. In addition, owing to their lower operating frequency and near-field coupling distance, passive LC sensors can achieve highly energy-efficient transmission and highly efficient data collection in harsh environments. Recently, numerous studies have investigated passive LC sensors to measure pressure, temperature, and humidity in harsh environments. Furthermore, the development of wireless passive measurement technology can address issues (such as unstable ohmic contacts or inadequate packaging) that are particularly problematic in environments involving high temperature, high rotation, sealed enclosures, or biological tissues.

**Table 1:** Sensor types, sensor characteristics, and harsh-environment applications.

Category	Characteristics	Typical Applications
Biomedical sensors	*In vivo*	Pressure monitoring in eyes, blood vessel, and abdominal aortic aneurysm, *etc.*
High-temperature sensors	−55 to 1500 °C	Aircrafts, engines, steel process control, and environmental monitoring
Humidity and gas sensors	Resistant to corrosion and high humidity	Harmful component monitoring in chemical plants, fuel cells, and packaged foods, *etc.*

This paper summarizes the wireless measurement principle, model design, and latest research on passive LC sensors applied to monitor pressure, temperature, humidity, and harmful gases. Development trends are also predicted.

## 2. Wireless Measurement Principles and Sensor Design Model

**Figure 2:**
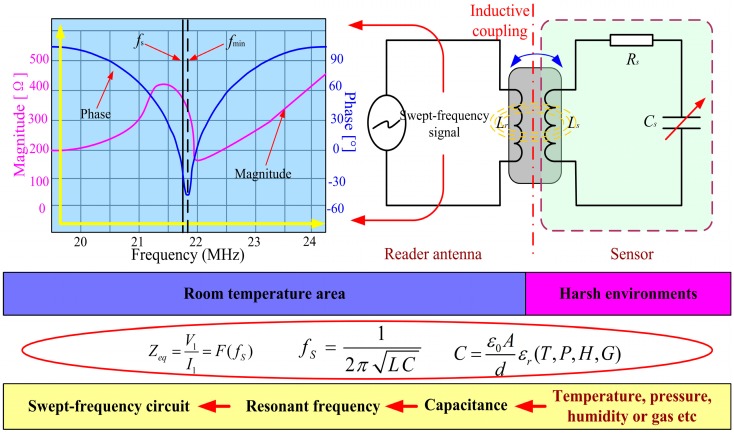
Principles and equations for wireless sensor measurements.

The lumped circuit model of a wireless passive LC sensor is equivalent to an LC circuit, where C is the sensitivity capacitance (Figure 2). The resonant frequency of a sensor drifts according to variations in the measured parameters (e.g., temperature, pressure, and humidity). When the swept-frequency signal generated by the measurement antenna passes over the resonant frequency of the sensor, the impedance characteristics (such as phase and magnitude) as seen by the antenna can be extracted because of the coupling link between the measurement antenna and the sensor. Thus, wirelessly measuring the resonant frequency of the sensor becomes possible. Figure 2 shows the process of performing wireless sensor measurements: *f*_s_ is the sensor resonant frequency, and *f*_min_ is the frequency of the lowest point of the phase angle as seen by the measurement antenna [8].

(1) $$f_{\min}=f_{S}(1+\frac{k^{2}}{4}+\frac{1}{8Q^{2}})$$

where *k* is the coupling coefficient between the inductance coils of the antenna and the sensor, and *Q* is the quality factor of the sensor. If *k* is sufficiently low and *Q* is sufficiently high, the resonant frequency of the sensor in a harsh environment can be determined by monitoring the variation in *f*_min_. Thus, the sensor is an LC resonant circuit with variable capacitance that can wirelessly communicate with an external measurement antenna.

The sensor can be integrated into a measurement system by magnetically coupling it with a loop antenna with inductance *L*_r_ so that the magnitude and phase of the sensor impedance can be wirelessly retrieved. Analysis of the overall circuit demonstrates that the impedance can be obtained as follows [9]:

(2) $$Z_{eq}=\frac{V_{1}}{I_{1}}=j2\pi fL_{r}\left[1+k^{2}\frac{\left(\frac{f}{f_{s}}\right)}{1-{\left(\frac{f}{f_{s}}\right)}^{2}+\frac{1}{Q_{s}}j\frac{f}{f_{s}}}\right]=F(f_{s})$$

where *V*_1_ and *I*_1_ are the voltage and current, respectively, of the measurement antenna, *L*_r_ is the inductance of the antenna, and *f* is the excitation frequency.

The quality factor of a sensor describes the energy transmission efficiency of a given passive LC resonant sensor. An inductor with higher quality factor is beneficial because it can acquire more power via inductive coupling and can more effectively transmit data to an external receiver. To improve the performance of spiral inductors, designers have evaluated spiral inductors with square, hexagonal, octagonal, and circular cross sections. In 1928, Wheeler H.A. proposed a formula for calculating the approximate inductance of a square planar spiral inductor as follows [10]:

(3) $$L\approx 2.34u_{0}\frac{n^{2}d_{avg}}{1+2.75\rho }$$

where *d*_avg_ = ((*d*_out_ + *d*_in_)/2) is the average diameter of the inductance coil and ρ = ((*d*_out_ − *d*_in_)/(*d*_out_ + *d*_in_)) is the fill ratio. *d*_out_ is the coil outer diameter, *d*_in_ is the coil inner diameter, *u*_0_ is the permeability of free space, and *n* is the number of turns of the inductance coil. In 1999, Lee T. proposed an accurate formula to calculate the inductance of a square planar spiral inductor [11].

(4) $$L=\frac{u_{0}n^{2}d_{avg}c_{1}}{2}(\ln(c_{2}/\rho )+c_{3}\rho +c_{4}\rho^{2})$$

where *c*_1_, *c*_2_, *c*_3_, and *c*_4_ are constants determined by the shape of the inductance coil. If the construction area, line width, line thickness, and materials of the inductance coil are given, the quality factor can be improved by improving the ratio of the inner and outer diameters of the inductance coil. The quality factor can be expressed as follows [12]:

(5) $$Q=\frac{wud_{out}hl(1-\alpha )}{4\pi \rho_{r}s}(\ln\frac{1+\alpha }{1-\alpha }+0.2235\frac{1-\alpha }{1+\alpha }+0.276)$$

where *w*, *u*, *h*, *l*, ρ_r_, and *s* are the angular frequency of the sensor, relative permeability of air, height of the inductance coil, width of the inductance coil, resistivity of the inductance coil, and spacing between adjacent turns of the inductance coil, respectively. *a* is the ratio of the inner and outer diameters of the inductance coil, which can be improved to achieve a maximum *Q* value.

## 3. Research Status of Wireless Passive LC Sensors for Harsh Environments

### 3.1. High-Temperature Environments

#### 3.1.1. High-Temperature Sensors for Pressure Measurements

**Figure 3:**
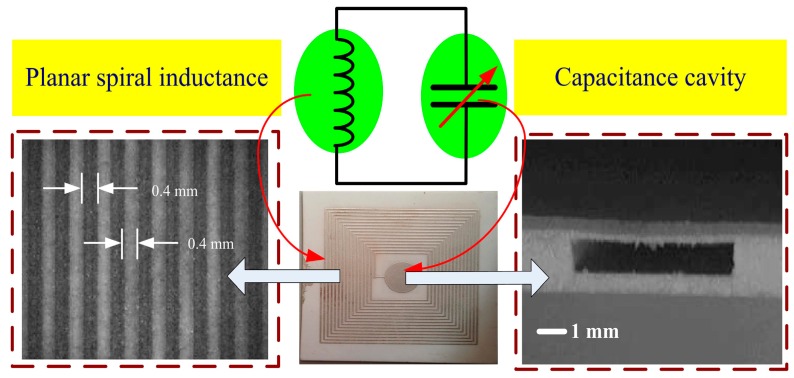
Wireless passive LC ceramic pressure sensor.

For silicon or silicon-on-insulator materials, the mechanical properties easily deteriorate, and the leakage current across the junctions drastically changes with increasing temperature, which limits the operating temperature range of the sensors based on these materials. Ceramic is a good insulator and is chemically stable in high-temperature environments, which enables sensors based on ceramics capable of wide application in high-temperature environments. The fabrication process of high-temperature passive LC ceramic pressure sensors is simple, which includes milling, tape casting, cutting, punching, screen printing, stacking, lamination, and firing. A typical structure of an LC ceramic pressure sensor is shown in Figure 3. In 1999, the team of Professor M. G Allen at the Georgia Institute of Technology pioneered and developed a wireless passive LC pressure sensor based on low-temperature co-fired ceramic (LTCC) technology. Experimental results demonstrated that the average accuracy and sensitivity of this sensor are 24 mbar and −141 kHz/bar, respectively, at 400 °C within a pressure range of 0 to 7 bar [13,14,15]. In 2009, a research team in Novi Sad, Serbia, proposed a better structural model. The improved structural design and the use of LTCC material made this embedded structural sensor suitable for high-temperature and chemically aggressive environments [16,17]. In 2013, researchers at the North University of China, which has been committed to developing wireless, high-temperature passive LC ceramic pressure sensors, introduced a unique screen-printing process involving a sacrificial layer of ESL 4900 to prevent deformation of a capacitive embedded cavity during lamination and sintering. This approach improved the flatness of the sensor cavity and led to better performance. The sensitivity of LTCC pressure sensors can reach −344 kHz/bar [18], the sensitivity of sensors based on high-temperature cofired ceramic (HTCC) can reach 860 Hz/bar [19,20], and sensors based on zircon ceramics can maintain stable operation at 800 °C for 30 min [21]. In 2014, researchers at the North University of China proposed improved structural models based on the LTCC, HTCC, and thick-film integrated technologies to improve the performance parameters (e.g., sensitivity, pressure range, operating temperature range, and coupling distance between the sensor and antenna) in harsh environments. Experimental results demonstrated good performance of these improved sensors, namely, sensitivity of up to 13 kHz/kPa, maximum working distance of up to 5 cm, maximum working pressure of up to 60 bar, and consistently low repeatability and hysteresis errors [22,23,24,25,26].

#### 3.1.2. High-Temperature Sensors for Temperature Measurement

**Figure 4:**
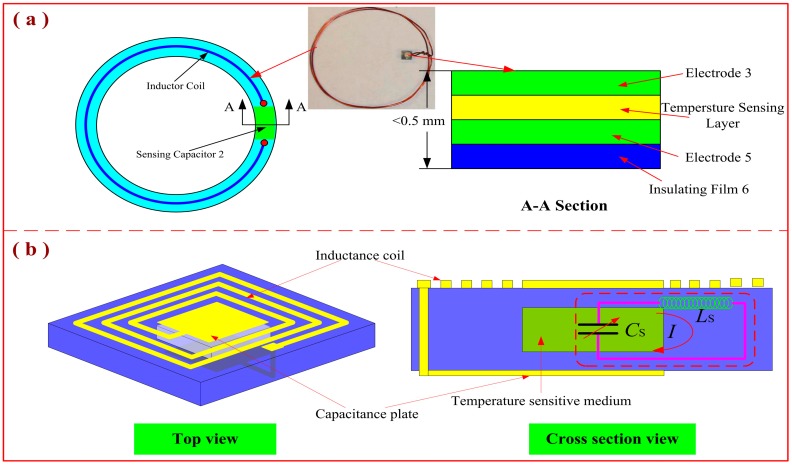
Wireless passive LC temperature sensors.

Accurate temperature measurement in harsh environments faces many challenges, but wireless passive LC sensors have become increasingly suitable for these harsh-environment applications. In 2009, J. Yi proposed a wireless passive temperature sensor (Figure 4a) to manufacture the components of a roller bearing. Ferroelectric ceramic was used for the sensor fabrication. With this sensor, the relative dielectric constant increases as the temperature increases. Experimental results demonstrated that the sensitivity of the sensor can be as high as 13 Hz/°C, which satisfies the temperature monitoring requirements for the manufacture of mechanical bearings [27]. In 2014, passive LC temperature sensors based on LTCC or HTCC were proposed. The typical structure of a passive LC temperature sensor is shown in Figure 4b. Initially, a wireless passive temperature sensor intended for harsh environments was realized using the LTCC technology. The dielectric of this sensor is based on ferroelectric ceramic, which exhibits temperature-sensitive permittivity. The sensor sensitivity is approximately −5.75 kHz/°C from room temperature to −430 °C and −16.67 kHz/°C from 430 to 700 °C [28]. Subsequently, to develop a sensor that can operate in higher temperature environments, Tan *et al.* proposed a wireless passive temperature sensor fabricated using multilayer HTCC tapes. The average sensitivity of this sensor, which exhibits good repeatability and reliability, can reach 5.22 kHz/°C with a temperature range from room temperature to 900 °C [29].

### 3.2. Biomedical Environments

**Figure 5:**
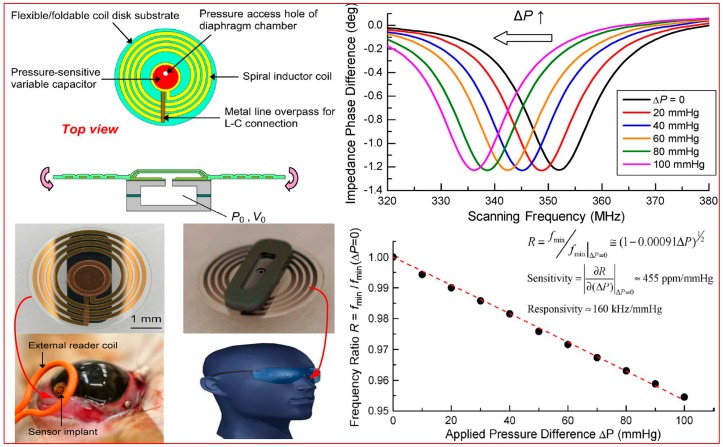
Implantable wireless passive LC pressure sensor for biomedical applications together with the experimental results.

To achieve pressure monitoring in, e.g., eyes, blood vessels, abdominal aortic aneurysm, or brain, researchers have explored passive wireless biomedical pressure sensors. Numerous studies have investigated pressure sensors for biomedical applications, and the principle of LC resonance coupling is currently attracting intense attention [30,31,32,33]. Researchers at the University of Liverpool proposed a passive wireless pressure sensor fabricated using micro-electromechanical systems (MEMS) technology and incorporating a biologically compatible waterproof material. The sensitivity of this sensor can reach 42.85 kHz/mmHg. Professor Y.C. Tai from the California Institute of Technology also demonstrated a wireless passive LC intraocular pressure (IOP) sensor based on parylene materials. The actual response of the sensor was evaluated in the eye of a rabbit. The results indicate that the sensitivity of the sensor can reach 205 kHz/mmHg with 1-mmHg resolution. Preliminary implementation of this sensor system has already been performed (Figure 5). In 2013, Professor G. Z. Chen proposed a capacitive contact lens sensor for continuous non-invasive IOP monitoring, which could track pressure variations over time with minimal lag. In addition, the IOP sensitivity of the sensor can reach up to 200 ppm/mmHg, and the linearity of the sensor is good, which can be up to 0.997, obtained in a porcine-eye test. In 2014, Professor J.B. Wang proposed a new differential transduction circuit based on the LC mutual inductance detection mechanism for gastrointestinal pressure monitoring, which is composed of a sensor capsule and a detection unit. The micro-pressure system can realize long-term monitoring stability, and the sensitivity can reach up to 0.2491 kHz/kPa [34,35,36].

### 3.3. Harmful Components of Harsh Environments

#### 3.3.1. Humidity Monitoring

Wireless passive LC sensors offer a highly advantageous ability to provide humidity monitoring in some special environments such as in concrete structures and food packaging. In these sensors, a humidity-sensitive material such as polyimide is deposited onto an interdigital capacitance. When the humidity-sensitive material absorbs moisture from the surrounding environment, the relative dielectric constant of the sensitive material changes, and thus, the interdigital capacitance changes. Consequently, the resonant frequency of the sensor can be determined by an external measurement antenna. An LTCC passive sensor for monitoring the humidity of building materials was proposed by G.M. Stojanovi *et al.* [37]. LTCC materials were used to fabricate the sensor, and as a result, the sensor exhibited good temperature stability. E.L. Tan *et al.* proposed a humidity sensor for *in situ* monitoring of the quality of dry packaged foods such as baked and fried snacks and cereals [38]. Because the dielectric constant of the test samples was much lower than that of water, higher moisture content in the samples caused the capacitance of the sensor to increase, leading to a decrease in the resonant frequency of the sensor. This change in the resonant frequency can be detected by a measurement antenna, and thus, the system can monitor humidity changes inside the package that could lead to degradation in the food quality.

#### 3.3.2. Gas Monitoring

In 2002, K.G. Ong at Pennsylvania State University proposed a wireless passive LC gas sensor [39,40,41]. A multiwalled carbon nanotube was used for the gas-sensitive dielectric material of the sensor, which can monitor CO and NH_3_ levels. When the SiO_2_ of the multiwalled carbon nanotube absorbs gases, the dielectric constant and conductivity of the material change according to the gas composition. This change leads to a change in the resonant frequency of the sensor, which is wirelessly detected by an external measurement antenna. This sensor can be used to monitor long-term harmful gas concentrations in a sealed opaque container. Professor Y. Ling from the University of Massachusetts Lowell fabricated a passive LC sensor based on single-walled carbon nanotube materials to monitor NH_3_ levels. The average sensitivity of this sensor was 0.76%/10^−6^ [42].

## 4. Development Trends of Passive LC Sensors

**Figure 6:**
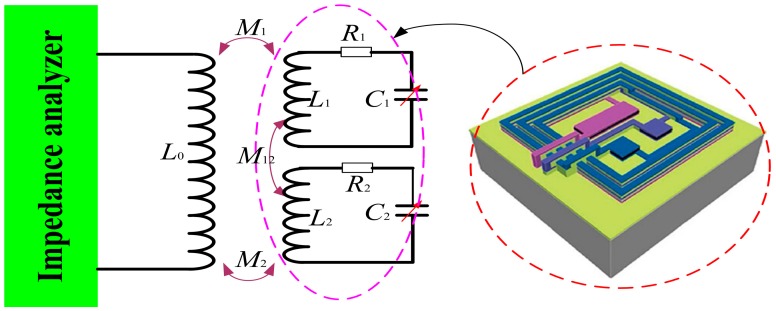
Model of a passive LC sensor capable of simultaneously measuring multiple parameters.

With the development of multifunction sensors, wireless passive LC sensors have also gradually integrated multiple measurement functions. The team of Professor Huang at Southeast University has investigated passive integrated sensors for Internet applications. Figure 6 shows an integrated sensor proposed by the researchers at Southeast University. The sensor is based on MEMS technology. It can simultaneously measure multiple parameters (such as temperature and pressure) and operate at temperatures up to 100 °C [43]. However, measuring multiple parameters using a single sensor, especially in harsh-environment applications such as high-velocity aeronautical measurements or internal-engine monitoring, suffers from many significant disadvantages. For the multifunction-sensor design, multiple coupling fields are needed to measure multiple parameters. The sensor design is complex compared with that of the signal coupling fields. Fabrication of the sensor faces many challenges in terms of the selection of dielectric materials and fabrication of the sealed cavity. For the measurement of multifunction sensors, because of the mutual inductance among multiple parameters, precise readout of multiple parameters requires a complex decoupling method. In addition, many other challenges exist, including poor adaptability, complex installation, and high cost, *etc.*, and current passive LC sensors exhibit some degree of temperature drift. Therefore, temperature compensation based on a high-temperature testing process is crucial to obtain accurate high-temperature data. Consequently, future development in this field will focus on passive LC sensors that can effectively and simultaneously measure multiple parameters in harsh environments using a single device.

In addition, passive LC sensors can benefit from new materials that provide improved sensitivity and wider operating ranges. The measurement system is also an important area for future development. Passive LC sensors will become even more suitable for harsh-environment applications when combined with smaller and more effective measurement system.

## 5. Conclusions

Passive LC resonant sensors do not require wired connections or external power supplies, and thus, they can readily be applied to complex measurements in harsh environments. Furthermore, these sensors can realize high-speed data collection and perform highly efficient energy transfer within smaller frequency ranges and working distances. Thus, research into passive LC sensors has found them advantageous for measuring various physical, chemical, and biological parameters. With expanding needs for effective sensors and the development of micro/nano processing technology, we expect that future research will employ passive LC designs in the development of monolithic sensors capable of measuring multiple parameters.
